# The effect of the COVID-19 pandemic on the mental health of patients with rheumatic diseases

**DOI:** 10.3389/fpsyt.2022.1007101

**Published:** 2022-10-24

**Authors:** Hiba Ramdani, Othman Moueqqit, Abdelilah Lahmar, Jucier Gonçalves Júnior, Estelita Lima Cândido, Samuel Katsuyuki Shinjo

**Affiliations:** ^1^Faculty of Medicine and Pharmacy of Oujda, Mohammed First University of Oujda, Oujda, Morocco; ^2^Division of Rheumatology, Faculdade de Medicina FMUSP, Universidade de São Paulo, São Paulo, Brazil; ^3^Faculdade de Medicina, Universidade Federal do Cariri (UFCA), Barbalha, Brazil

**Keywords:** autoimmune diseases, COVID-19, mental health, pandemic, rheumatic diseases

## Introduction

In addition to patients with autoimmune rheumatic diseases (ARDs) with a significant rate of psychiatric disorders, the real effect of the COVID-19 pandemic on the mental health of patients with ARDs, as well as the risk factors, has not yet been properly mapped (1–3).

Previous studies have shown that patients with psychiatric illnesses have increased levels of pro-inflammatory cytokines (e.g., interleukins 1 and 8 and tumor necrosis factor-alpha—TNF-alpha), which are known to be intrinsically involved in the etiopathogenesis of ARDs (4) and the response to viral agents such as SARS-CoV-2. In parallel, the etiologic agents of previous pandemics presented several neuropsychiatric symptoms, such as psychoses, encephalopathies, neuromuscular dysfunction, and demyelination (5). Epidemiological studies have shown poorer mental health scores, increased incidence of psychiatric illnesses (2, 6, 7), and negative symptoms (8) among patients with ARDs as the pandemic progressed. However, findings in the literature are conflicting (9, 10).

Thus, we aimed to discuss possible risk factors for poor mental health in patients with ARDs during the COVID-19 pandemic.

## Discussion

**Figure 1:**
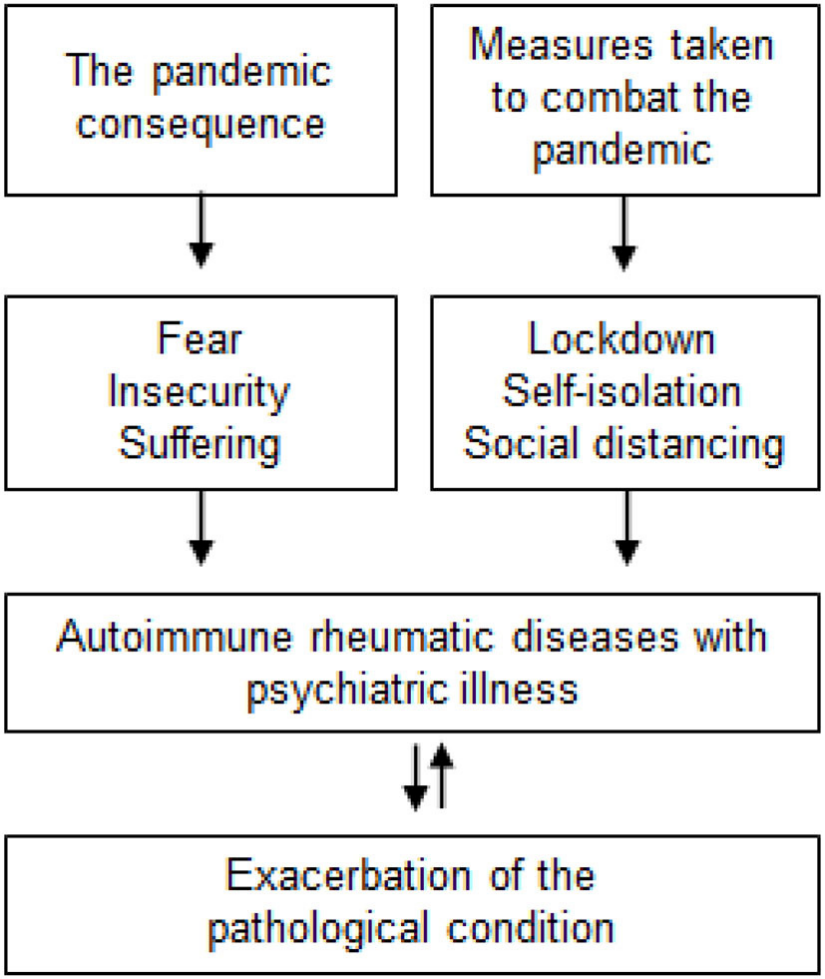
A diagram illustrating the factors contributing to the exacerbation of pathological condition in autoimmune rheumatic disease patients with psychiatric illness.

The first factor to consider is the high rate of psychiatric illnesses in patients with ARDs. The pathologies can be exacerbated by the status of fear, insecurity, and suffering that the pandemic has brought, as well as measures to combat it such as lockdowns, self-isolation, and social distancing (1, 2) (Figure 1).

Ziadé et al. reported negative mental health consequences of COVID-19 precautions in 73% of the participants with ARDs (3). However, the number of patients reporting suspected depression has increased significantly after the COVID-19 pandemic began (2). Literature survey has shown that because mental illnesses and ARDs both produce pro-inflammatory cytokines, their co-occurrence has a pathophysiological explanation. Moreover, neuropsychiatric manifestations in the context of COVID-19 are widely recognized, but no etiopathogenic mechanisms have been elucidated (4, 5). A study performed on 1,800 patients with 15 types of ARDs showed that 57.3 and 45.9% were at high risk for anxiety and depression, respectively (6). A survey conducted on 307 patients with ARDs, in Morocco, showed a significant prevalence of undiagnosed depression, anxiety, and insomnia symptoms. It is noteworthy that in this study major depression was associated with the worsening of rheumatic disease (7). An Italian study of 507 patients with ARDs showed that female sex, younger age, being overweight or obese, fear of loss of income, and treatment for psychiatric pathologies were associated with worse levels of stress. Insomnia was observed in 375 patients with arthritis and was associated with older age, previous psychiatric illness, and having been infected by COVID-19 (11).

However, it is important to note that studies carried out in Holland (9) and the United States (10) showed that patients with ARDs had negative symptom rates that were very close to those of the general population, as well as good coping strategies. Nevertheless, these studies were conducted in developed countries, where health conditions and access to basic subsistence services are better, and the samples were small.

Because patients with ARDs are in a higher risk group (e.g., due to using immunosuppressive drugs, or having pulmonary impairment) for more severe forms of COVID-19, the second factor that should be evaluated is whether they have greater psychic overload than the general population. A case-control study of 360 participants, divided into a patient group of 180 people with ARDs and a control group of 180 people without ARDs, determined that patients with ARDs experience more discomfort and panic symptoms in the form of anger, irritability, and insomnia (8). However, the data were not homogenous. Koppert et al. (9) reported that levels of mental wellbeing were not reduced in patients with ARDs to those in the control group.

The third factor to be considered is the negative effect of the pandemic on global rheumatological care, which has been well-documented in the literature. This impact has been manifested as numerous disruptions among patients, canceling appointments or switching to telemedicine, difficulties accessing emergency care, and fear of catching infections due to hospital visits (12). Disruptions to routine and emergency care caused by the pandemic had a negative effect on the mental and physical health of rheumatic patients (12) (Figure 1).

Furthermore, the temporary reduction in the availability of care resulted in not only a significant rise in physical damage but also in psychological harm and medical insecurity in many patients (12). Another prospective study found that changes in clinical care as a result of lockdowns were linked to worse disease outcomes, which had a clear influence on mental health (13).

Fourth, it is interesting to highlight the inability to perform regular physical exercise during the pandemic, which is a criterion standard for the treatment of various symptoms of rheumatic diseases (e.g., fatigue, muscle pain, and insomnia) withdraw (2, 14, 15). Recent studies have suggested that increasing physical activity and exercise may improve symptoms and reduce the effect of systemic manifestations of rheumatoid arthritis. Therefore, a lack of physical activity has been largely associated with the worsening of global assessments, emotional stress, depression, pain, and fatigue (15). Limited access to gyms, sports facilities, and equipment, as well as lack of desire, fatigue, fear of worsening pain, and misleading information about safety, were among the reasons for lower physical activity during COVID-19, which consequently jeopardized mental health and wellbeing of those already at risk of low physical activity and high levels of physical inactivities, such as individuals with rheumatoid arthritis (15) (Figure 1).

## Conclusion

In short, because mental illnesses are underdiagnosed and often untreated, psychological follow-up care for patients with ARDs is crucial. Although there is disagreement in the literature regarding the actual effect of the COVID-19 pandemic on the mental health of patients with ARDs, a biopsychosocial approach can help clinicians understand the bidirectional relationship between mental health problems and rheumatic diseases, which can contribute to full assessment and comprehensive treatment to reduce the burden of disease.

Research aimed at mapping the mental health, lifestyle habits, and quality of life of patients with various ARDs before and after the pandemic, can be valuable for planning public policies and contingency plans in situations such as pandemics.

## Author contributions

All authors listed have made a substantial, direct, and intellectual contribution to the work and approved it for publication.

## Funding

This work was supported by Pró-Reitoria de Pesquisa, Pós-Graduação e Inovação, and Programa de Desenvolvimento Regional Sustentável (PRODER)—Universidade Federal do Cariri (UFCA) and Faculdade de Medicina FMUSP to SS.

## Conflict of interest

The authors declare that the research was conducted in the absence of any commercial or financial relationships that could be construed as a potential conflict of interest.

## Publisher's note

All claims expressed in this article are solely those of the authors and do not necessarily represent those of their affiliated organizations, or those of the publisher, the editors and the reviewers. Any product that may be evaluated in this article, or claim that may be made by its manufacturer, is not guaranteed or endorsed by the publisher.
